# 17β-estradiol and estrogen receptor alpha protect mouse ovarian follicle development by repressing atresia

**DOI:** 10.1016/j.isci.2025.111846

**Published:** 2025-01-20

**Authors:** Eri Ueno, Mitsuya Watanabe, Yoshiko Kondo, Naomi Nakagata, Toru Takeo, Satohiro Nakao, Katsueki Ogiwara

**Affiliations:** 1 Laboratory of Reproductive and Developmental Biology, Faculty of Science, Hokkaido University, Sapporo 060-0810, Japan; 2 Division of Reproductive Biotechnology and Innovation, Center for Animal Resources and Development, Institute of Resource Development and Analysis, Kumamoto University, 2-2-1 Honjo, Chuo-ku, Kumamoto 860-0811, Japan; 3 Division of Reproductive Engineering, Center for Animal Resources and Development, Institute of Resource Development and Analysis, Kumamoto University, 2-2-1 Honjo, Chuo-ku, Kumamoto 860-0811, Japan

**Keywords:** Endocrinology, Rodent reproduction, Molecular physiology

## Abstract

Mice administered an inhibin antiserum, equine chorionic gonadotropin (eCG) mixture called CARD HyperOva (OVA)/human chorionic gonadotropin (hCG), ovulate more oocytes than those administered eCG/hCG. In this study, the mechanism by which more oocytes are ovulated was investigated. Apoptotic cells were not observed in ovaries 24 and 48 h after OVA injection, and INHBA expression was absent in the growing follicles with apoptotic cells, whereas granulosa cells in follicles expressing INHBA also expressed estrogen receptor α (ESR1). ESR1 and CYP19A1 expression and 17β-estradiol (E_2_) in sera significantly increased in OVA-injected mice. *Esr1* and *Cyp19a1* expression and E_2_ concentration increased in ovaries cultured with activin A. The ovulation number increased in mice administered diethylstilbestrol. Taken together, these results suggest that ESR1 and E_2_ are involved in the inhibition of follicular atresia, which increases ovulation and oocyte number. This study may provide valuable information on the molecular mechanisms underlying mouse follicle selection.

## Highlights

• Apoptotic cells were not observed in ovaries 24 and 48 h after OVA injection
• Granulosa cells in follicles expressing INHBA and ESR1 were TUNEL-negative
• *Esr1* expression and E_2_ concentration increased in ovaries cultured with activin A
• ESR1 and E_2_ are involved in the inhibition of follicular atresia

## Graphical abstract

**Figure:**
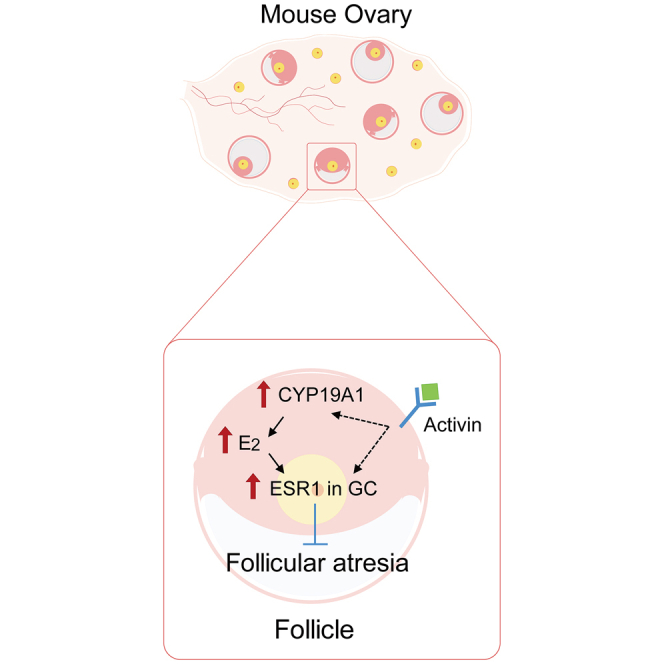

## Introduction

Follicular selection is a unique and physiologically important process by which dominant follicles containing high-quality oocytes are selected from the recruited cohort or wave of growing follicles.^1^^,^^2^^,^^3^^,^^4^^,^^5^ In mammals, some follicle growth is initiated from a pool of primordial follicles in the ovary via stimulation by an unknown growth-hormone-like hormone.^6^ However, most follicles are lost owing to atresia, and only a few follicles reach the preovulatory stage and ovulation.^7^^,^^8^ A major process responsible for follicular loss in the ovary is follicular atresia, which is initiated by apoptosis of granulosa cells (GCs).^9^ To date, many studies have reported candidate factors that are thought to be involved in follicle selection and have proposed models for the mechanism of follicle selection in some species, particularly mono-ovulation animals.^10^^,^^11^ Gonadotropins such as follicle-stimulating hormone (FSH) and luteinizing hormone (LH) are thought to be the most important factors regulating the promotion and/or suppression of follicular growth and/or follicular atresia.^12^ Many growth hormones are reported to be involved in follicle growth and survival, including insulin-like growth factor^13^ and members of the transforming growth factor β superfamily (inhibins and activins).^14^^,^^15^ Although the follicle selection process has been investigated in many studies, its complete mechanism remains unknown.

Inhibin A, which is a heterodimer of inhibin α (INHA) and inhibin βa (INHBA), is involved in the inhibitory regulation of FSH secretion from the anterior pituitary gland.^16^ Inhibins and activins form dimers and are secreted from the cells.^17^ Negative regulation of FSH secretion by inhibin A is thought to be important for follicle selection.^5^ Injection of inhibin antiserum (IAS) into rats neutralizes the function of inhibin A and causes an increase in FSH, resulting in the induction of follicle growth and an increase in the number of ovulations.^18^ In addition, IAS injections into hamsters, rats, guinea pigs, cows, and mares increased the number of ovulated oocytes.^18^^,^^19^^,^^20^^,^^21^^,^^22^

Super-ovulation is a reproductive technology used to increase the number of ovulated oocytes. Approximately 20–30 oocytes are ovulated in mice administered equine chorionic gonadotropin (eCG)/human chorionic gonadotropin (hCG),^23^ whereas we previously reported that over 100 oocytes are ovulated in mice administered a mixture of eCG and goat anti-mouse IAS called CARD HyperOva (OVA), followed by hCG.^24^ However, the mechanisms of superovulation by OVA/hCG remain unknown.

This study aimed to elucidate the molecular mechanisms underlying follicle selection. Therefore, it is necessary to examine the molecular mechanisms underlying follicular atresia induction. The number of ovulated oocytes in mice treated with OVA/hCG was much higher than that in mice administered eCG/hCG,^24^ which led us to hypothesize that follicle selection was relaxed in mice treated with OVA/hCG. Our preliminary experiments showed that the number of apoptotic GCs in growing follicles decreased in OVA-treated ovaries compared with that in eCG-treated ovaries, suggesting that follicular atresia was suppressed in OVA/hCG-injected mice. If the molecular mechanism by which follicular atresia is inhibited can be elucidated, it is likely that the mechanism of follicle selection can be described based on this information. Therefore, we analyzed the molecular mechanisms by which the number of ovulated follicles increased in mice with administered OVA/hCG.

## Results

### Detection of apoptotic cells in the ovaries of mice administered eCG or OVA

**Figure 1:**
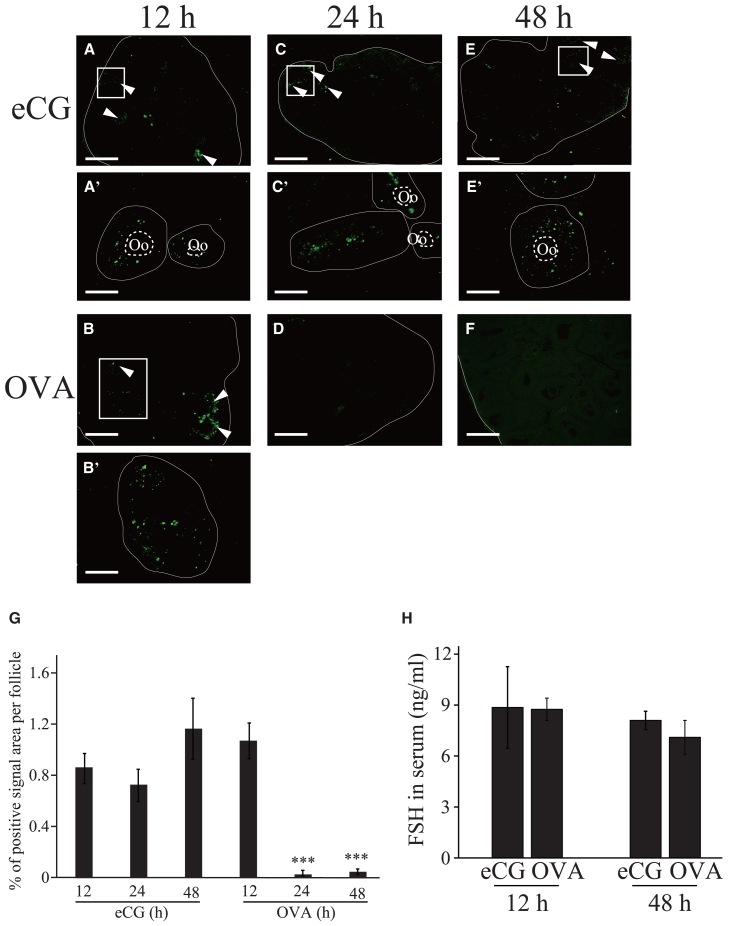
Detection of apoptotic cells and FSH levels in the ovaries and sera of mice administered eCG or OVA (A–F) Apoptotic cells were detected using the TUNEL assay with sections prepared from the ovaries from mice 12 h (A and B), 24 h (C and D), and 48 h (E and F) after eCG or OVA injection. Enlarged images in the box in (A–C) and (E) are shown in (A′–C′) and (E′), respectively. The area surrounded by the line and dotted line in (A′–C′) and (E′) represent follicle and oocyte (Oo), respectively. Arrowheads indicate the follicles with apoptotic GCs. Scale bars: 200 μm (A, C, E); 100 μm (B, D, F); 50 μm (A′, B′, C′, E′). (G) The apoptotic index was expressed as the percentage of the TUNEL-positive cells area per whole area of the follicle. ∗∗*p* < 0.01 (ANOVA and Tukey-Kramer test, *n* = 7). (H) Plasma FSH levels were measured using ELISA in mice 12 and 48 h after eCG or OVA injection (t test, *n* = 4).

A TUNEL assay was conducted to examine whether apoptosis occurred in the ovaries of mice administered eCG or OVA. Positive signals were detected in the GCs of some preantral and early antral follicles 12 h after eCG or OVA injection (Figures 1A, 1A′, 1B, and 1B′, arrowheads). Apoptotic cells were observed in some preantral and early antral follicles 24 and 48 h after eCG injection (Figures 1C, 1C′, 1E, and 1E′, arrowheads), whereas positive signals were significantly decreased in the ovaries 24 and 48 h after OVA injection (Figures 1 D, 1F, and 1G), suggesting that follicular atresia was suppressed in the ovaries between 12 and 48 h after OVA injection.

Serum FSH levels were measured using enzyme-linked immunosorbent assay (ELISA) 12 and 48 h after eCG or OVA injection. No significant difference was found in the serum FSH levels at 12 or 48 h after OVA injection compared with those after eCG injection (Figure 1G). These results suggested that the suppression of apoptosis was caused by the direct effect of OVA on the ovaries.

### Expression of INHA and INABA in the ovaries

**Figure 2:**
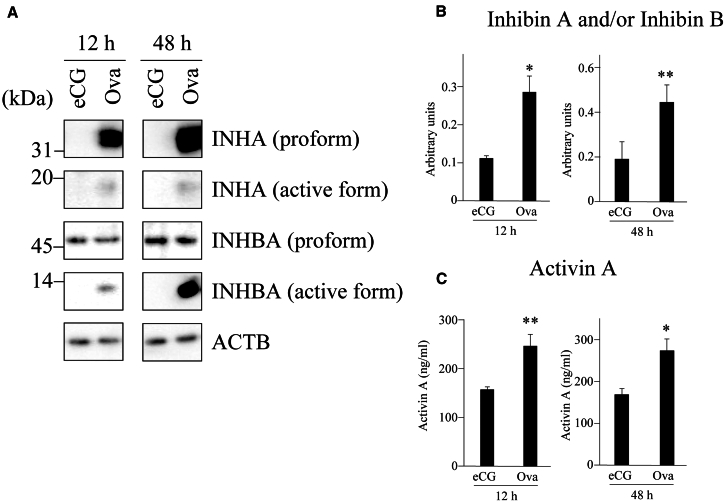
INHA and INHBA expression in ovaries and activin A and inhibin A levels in sera of mice administered eCG or OVA (A) INHA and INHBA expression were detected by western blotting using extracts prepared from the ovaries from mice 12 h and 48 h after eCG or OVA injection. (B and C) ACTB was used as a loading control. Plasma inhibin A and/or inhibin B (B) and activin A (C) levels were measured with ELISA using sera collected from mice administered eCG or OVA. ∗∗*p* < 0.01, ∗*p* < 0.05 (t test, *n* = 4).

The expression of inhibin subunit proteins in the ovaries of mice administered eCG or OVA was examined using western blotting. The expression levels of the pro and active forms of INHA in the ovaries of OVA-treated mice were increased at 12 and 48 h after treatment compared with those of eCG-injected mice (Figure 2A). The expression of the active form of INHBA in the ovaries of OVA-treated mice were also increased 12 and 48 h after injection, whereas the expression of its pro form did not change (Figure 2A). Inhibin A and/or inhibin B and activin A levels were measured in the sera of eCG- or OVA-injected mice using ELISA. Hormone levels were significantly increased at 12 and 48 h after OVA injection compared with those in eCG-treated mice (Figures 2B and 2C). The activin A levels were 1.57 and 1.62 times higher than those of mice 12 and 48 h after eCG injection, respectively. The inhibin A and/or inhibin B levels were 2.55 and 2.33 times increased in mice 12 and 48 h after OVA injection, respectively.

**Figure 3:**
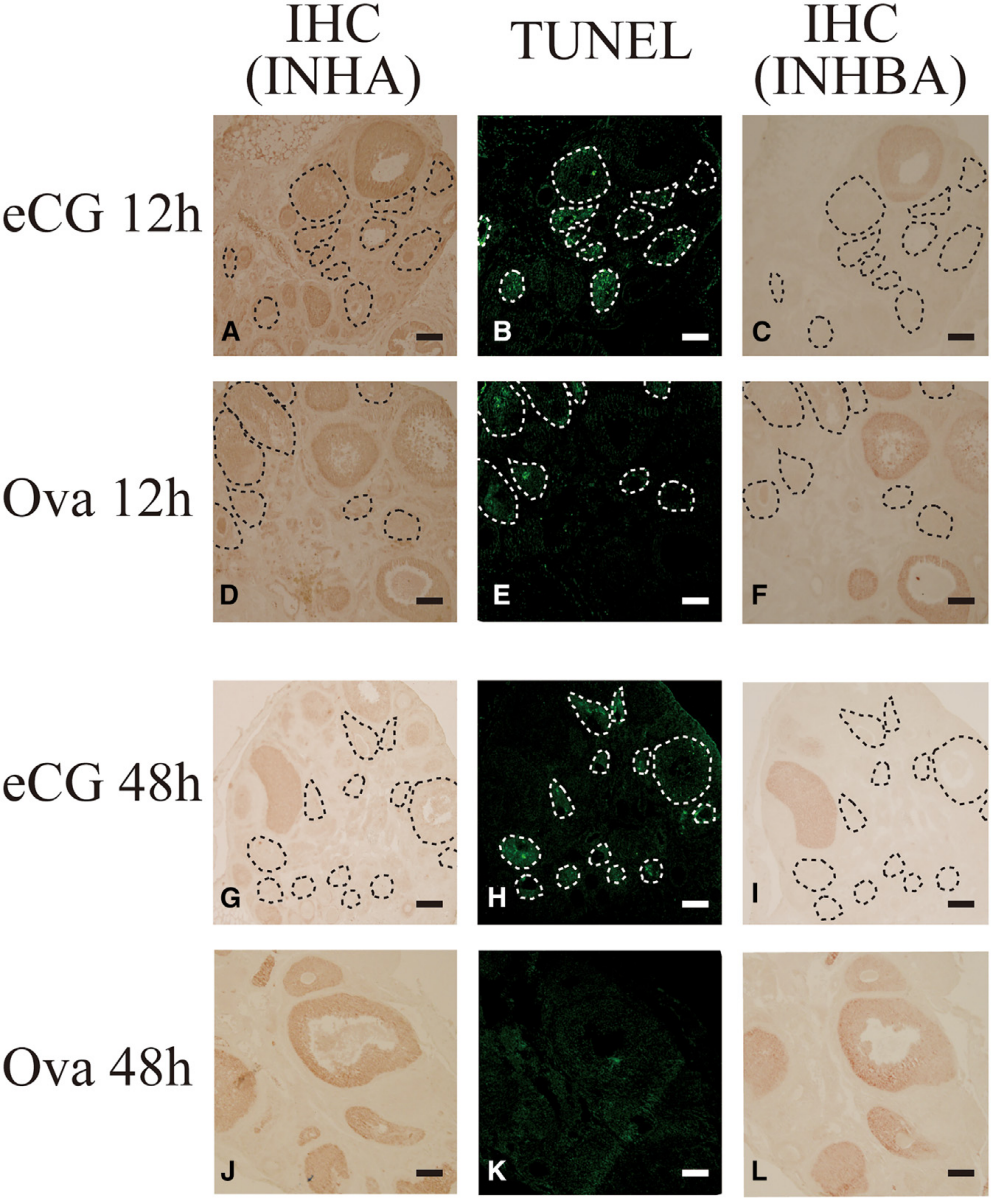
Detection of INHA and INHBA expression and apoptotic cells in ovaries of mice administered eCG or OVA (A–L) INHA (A, D, G, and J) and INHBA (C, F, I, and L) localization and apoptotic cells (B, E, H, and K) were detected using immunohistochemistry or TUNEL assay, respectively, using sequential sections prepared from mouse ovaries collected 12 and 48 h after eCG or OVA injection. Follicles with positive signals of TUNEL staining are indicated within white dotted lines. Follicles corresponding to those with positive signals of TUNEL staining are indicated within black dotted lines. Scale bars, 100 μm.

Immunohistochemical (IHC) analyses of INHA and INHBA, as well as TUNEL assays, were performed on sequential sections of the ovaries from eCG- or OVA-injected mice to determine whether apoptotic cells were present in follicles expressing INHA and/or INHBA. INHBA was localized in the growing follicles, especially in the antral follicles; however, no positive signals of TUNEL staining were observed in these follicles (Figure 3). INHBA-positive follicles also expressed INHA; however, some INHA-positive follicles were TUNEL-negative, whereas positive signals were detected in the others. No positive signals were observed when normal goat IgG (for INHA) or rabbit normal IgG (for INHBA) was used as negative controls, indicating that the signals were specific (Figure S1). These results suggest that INHBA is involved in the suppression of follicular atresia.

### Expression of *Cyp19a1*/CYP19A1 and 17β-estradiol (E_2_) levels in the sera and ovaries of OVA-injected mice

**Figure 4:**
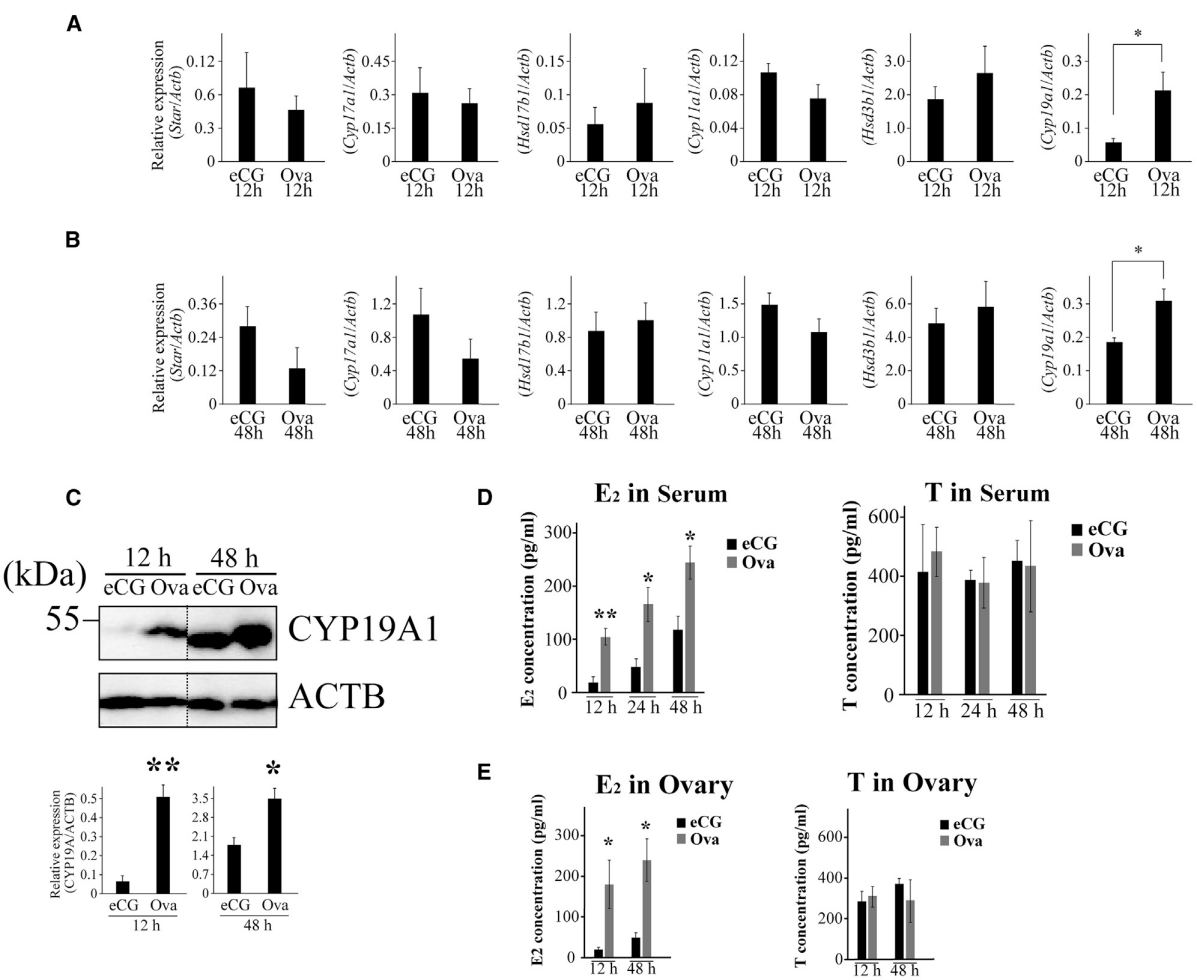
Expression of steroidogenic enzyme genes and E_2_ levels in ovaries and sera of mice administered eCG or OVA (A and B) RT-qPCR analysis of steroidogenic enzyme gene expression using cDNA prepared from the ovaries of mice collected 12 h (A) and 48 h (B) after eCG or OVA injection. ∗*p* < 0.05 (t test, *n* = 5). (C) CYP19A1 expression was detected by western blotting of extracts prepared from mouse ovaries collected 12 and 48 h after eCG or OVA injection. ACTB was used as a loading control (two upper panels). The signal intensity of the bands in the upper panels was quantified using densitometric analysis, and the ratio of CYP19A1 to ACTB expression was calculated as the relative expression (lower panels). ∗*p* < 0.05 and ∗∗*p* < 0.01 (t test, *n* = 4). (D) E_2_ and testosterone levels were measured in the sera collected from mice 12, 24, and 48 h after eCG or OVA injection using ELISA. ∗*p* < 0.05, ∗∗*p* < 0.01 (t test, *n* = 6). (E) E_2_ and testosterone levels were measured in the ovaries of mice collected 12 and 48 h after eCG or OVA injection using ELISA. ∗*p* < 0.05 (t test, *n* = 7).

Activin A is an INHBA homodimer that regulates the expression of genes involved in steroid hormone synthesis genes.^25^ We examined gene expression in ovaries of the OVA-injected mice. The mRNA expression of *Cyp19a1* (aromatase), which encodes a terminal enzyme in the steroidogenic pathway that converts androgens such as testosterone to estrogens such as E_2_,^26^ was significantly upregulated in the ovaries at 12 and 48 h after OVA injection compared with that in the eCG-injected mouse ovaries (Figures 4A and 4B). Similar results were obtained for CYP19A1 protein expression (Figure 4C). Next, E_2_ levels were measured in the sera and ovaries of the mice 12, 24, and 48 h after injection. Serum levels 12, 24, and 48 h after OVA administration were significantly higher than those in the sera of eCG-injected mice (Figure 4D). Similar results were obtained in experiments in which the levels were measured in the ovaries 12 and 48 h after OVA administration (Figure 4E). Conversely, no significant differences in testosterone levels were detected in any of the tested samples (Figures 4D and 4E).

### Expression of caspase genes in ovaries of eCG- or OVA-injected mice

**Figure 5:**
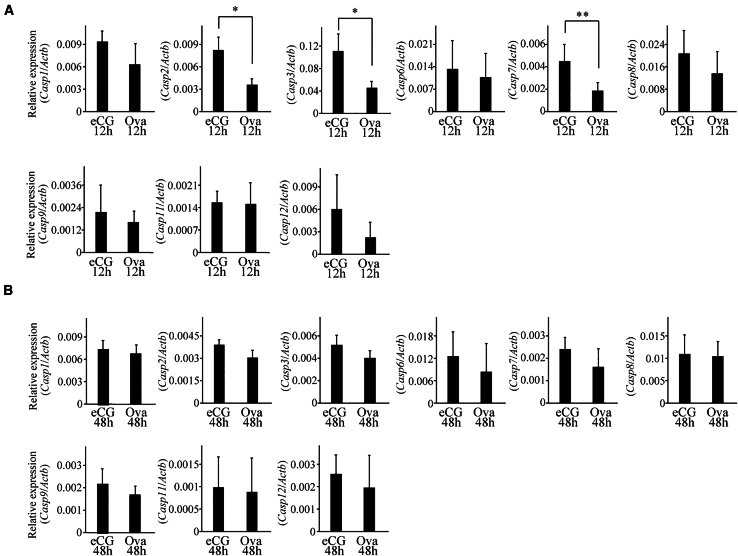
Expression of caspase genes in ovaries of mice administered eCG or OVA (A and B) RT-qPCR analysis of caspase gene expression using cDNA prepared from the ovaries of mice collected 12 h (A) and 48 h (B) after eCG or OVA injection. ∗*p* < 0.05, ∗∗*p* < 0.01 (t test, *n* = 5).

We examined the expression of caspase (*Casp*) genes in the ovaries of the eCG- or OVA-injected mice. Among the genes tested, the mRNA expression of *Casp 2*, *3*, and *7*, all of which are apoptotic caspases and not inflammatory caspases,^27^ was significantly decreased in the ovaries 12 h after OVA injection compared with that in the eCG-injected mouse ovaries, whereas no significant difference was observed 48 h after injection (Figures 5A and 5B). These results suggested that the decrease in the number of apoptotic cells in GCs (Figure 1) was caused by a decrease in the expression of the caspases.

### Effects of E_2_ or diethylstilbestrol injection on *in vivo* ovulation

**Figure 6:**
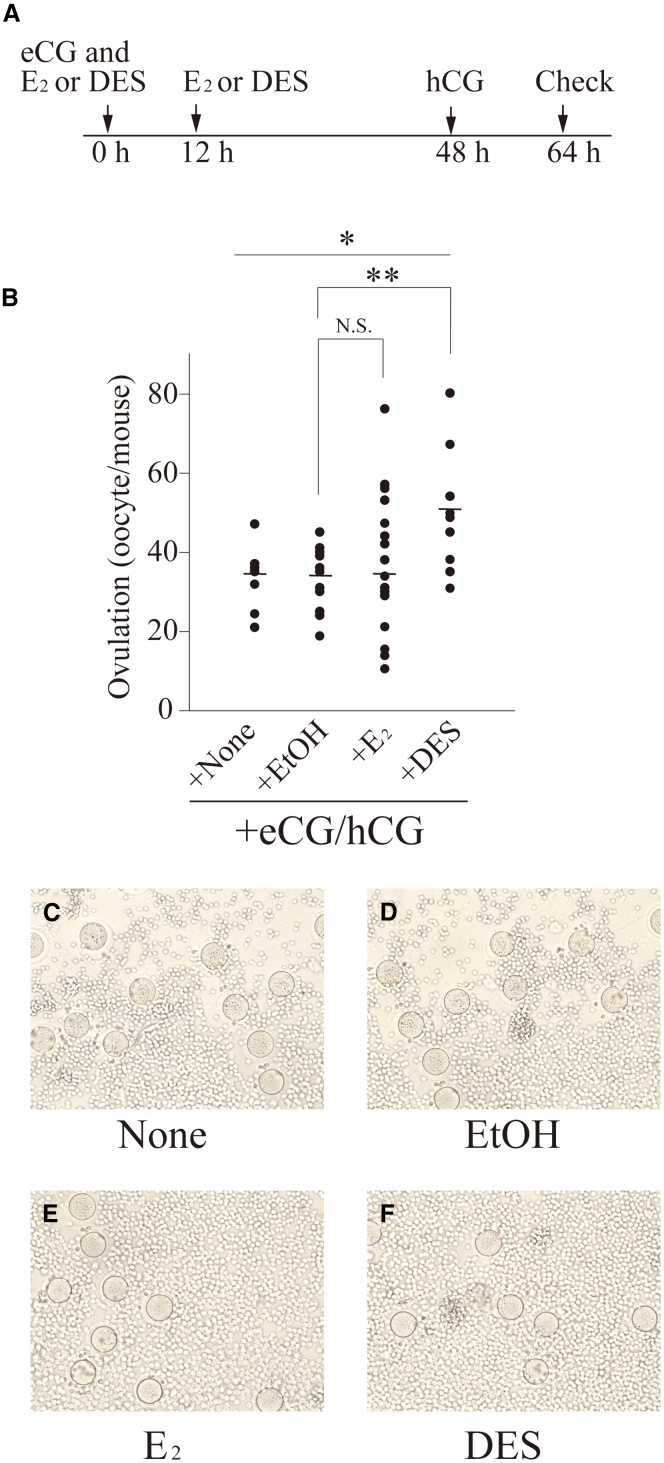
Effect of E_2_ or DES on the number of ovulated oocytes (A) The injection timing of eCG, hCG, E_2_, and DES is shown. (B) The number of ovulated oocytes in mice administered E_2_, DES, or EtOH together with eCG and hCG is shown. Each dot represents the number of ovulated oocytes from one individual mouse. The median value is indicated by a horizontal black line in each lane. ∗*p* < 0.05, ∗∗*p* < 0.01 (t test, *n* = 7–20). (C–F) The images of the ovulated oocytes are shown.

The ovulation rates of mice administered E_2_, diethylstilbestrol (DES), or ethanol (EtOH) together with eCG/hCG were examined to validate whether E_2_ is involved in follicle selection. The timing of the hormone injections is shown in Figure 6A. The number of ovulated oocytes tended to increase when E_2_ was injected into mice at 0 and 12 h after eCG injection compared with that in mice administered EtOH after eCG injection, although this increase was not significant (Figure 6B). In contrast, the number of ovulated oocytes increased significantly when DES, an artificial nonsteroidal analog of estradiol, was injected into mice. The mean values of the eCG, EtOH, E_2_, and DES groups were 33.0 ± 3.3 (median value: 35.0, *N* = 7), 33.6 ± 2.2 (median value: 35.0, *N* = 13), 36.0 ± 3.8 (median value: 35.5, *N* = 20), and 49.0 ± 5.6 (median value: 49, *N* = 9), respectively. The oocytes with normal morphology were observed (Figures 6C–6F). The developmental competence of the oocytes was evaluated using an *in vitro* fertilization method. Almost all oocytes were fertilized and developed normally, confirming that they were capable of fertilization and normal development (data not shown). The number of ovulated oocytes was checked in mice injected with E_2_ or DES together with OVA/hCG, but no significant difference was observed compared with the control (data not shown). These results suggest that E_2_ is involved in the suppression of follicular atresia, which increases the number of ovulated oocytes.

### Expression of *Esr1*/ESR1 and *Esr2* in the ovaries of OVA-injected mice

**Figure 7:**
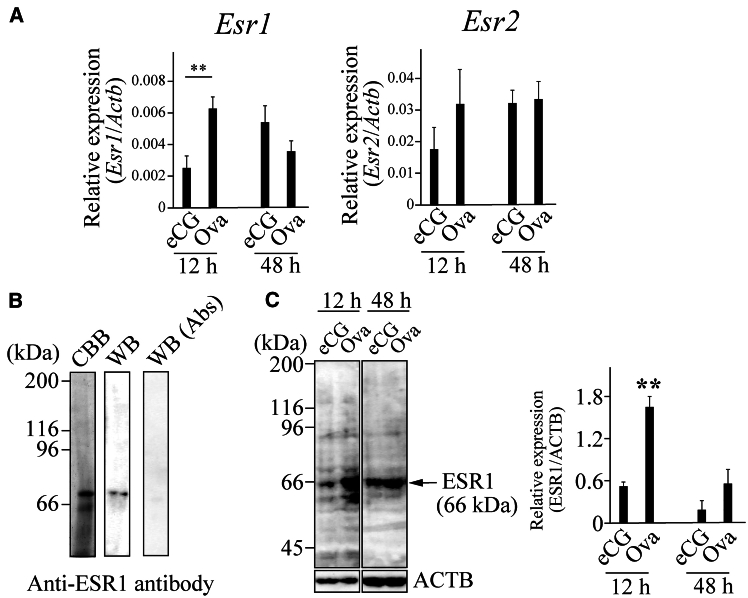
Expression of *Esr1*/ESR1 and *Esr2* in the ovaries of mice administered eCG or OVA (A) The expression of *Esr1* and *Esr2* was measured using RT-qPCR in the ovaries from mice administered eCG or OVA. ∗∗*p* < 0.01 (t test, *n* = 5). (B) The purity of an antigen against ESR1 was confirmed by SDS-PAGE/Coomassie Brilliant Blue (CBB) staining. The specificity of the anti-mouse ESR1 antibody was evaluated by western blotting using the antibody (WB) and an absorbed antibody (WB [Abs]). (C) Expression of ESR1 was examined by western blotting in the ovaries from mice administered eCG or OVA. ACTB is used as a loading control (lower panel). The signal intensity of the bands was densitometrically quantified, and the ratio of the relative expression of ESR1 to ACTB was determined (right panel). ∗∗*p* < 0.01 (t test, *n* = 5).

We examined whether *Esr1*/ESR1 and *Esr2* are expressed in the ovaries of OVA-injected mice. *Esr1* expression was significantly upregulated in the ovaries 12 h after OVA injection, whereas no significant difference was observed 48 h after injection (Figure 7A). No significant change in *Esr2* expression was detected in the ovaries 12 and 48 h after OVA injection compared with that after eCG injection (Figure 7A). We generated an ESR1-specific antibody to examine its protein expression in the ovaries. A single band was detected using SDS-PAGE/CBB staining when we assessed the quality of the purified antigen. A specific band was detected by western blot using an antibody at almost the same position as that observed by Coomassie Brilliant Blue (CBB) staining. No bands were observed on the western blots with the absorbed antibody, indicating that the antibody was specific (Figure 7B). ESR1 expression was significantly increased in the ovaries 12 h after OVA injection compared with that in the ovaries of mice administered eCG (Figure 7C). No bands were detected when western blotting was performed using normal rabbit immunoglobulin G (IgG) as a negative control (data not shown).

**Figure 8:**
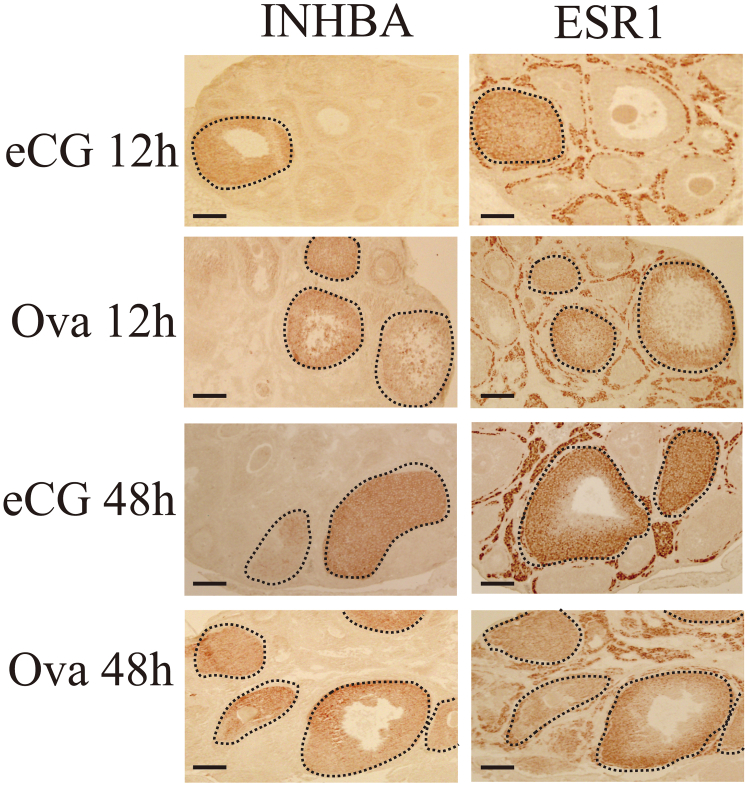
INHBA and ESR1 localization in the ovaries of mice administered eCG or OVA INHBA (left panels) and ESR1 (right panels) localization were detected using immunohistochemistry using sequential sections prepared from ovaries harvested 12 and 48 h after mice were administered eCG or OVA. Follicles positive for INHBA and ESR1 in the GCs are indicated within black dotted lines. Scale bars, 100 μm.

IHC was performed to examine ESR1 localization in ovaries after eCG or OVA injection. ESR1 was expressed in the theca cells of the follicles at various developmental stages (Figure 8). In addition, the receptor was detected in the GCs of growing follicles expressing INHBA (Figure 8, dotted lines). No positive signals were detected with normal rabbit IgG, indicating that the signals were specific to ESR1 (Figure S1). Taken together, these results suggest that ESR1, but not ESR2, which is expressed in GCs, is important for mediating follicular atresia and follicle selection.

### Regulation of *Cyp19a1* and *Esr1* expression and E_2_ production by activin A

**Figure 9:**
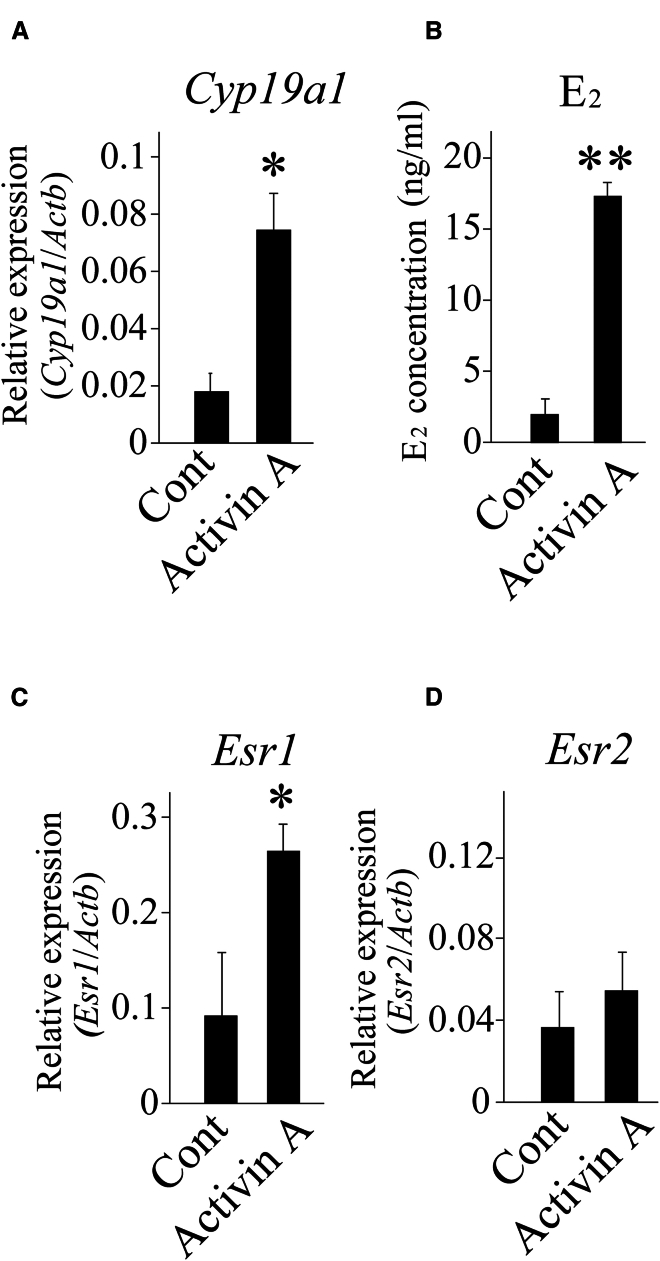
Effect of activin A on *Cyp19a1* and *Esr* expression and E_2_ production in cultured mouse ovaries (A, C, and D) The expression of *Cyp19a1* (A), *Esr1* (C), and *Esr2* (D) were examined using RT-qPCR using cDNA prepared from ovaries cultured with or without activin A. ∗*p* < 0.05 (t test, *n* = 3). (B) E_2_ levels in the culture media were measured using ELISA. ∗∗*p* < 0.01 (t test, *n* = 3).

We investigated whether activin A induced *Cyp19a1* and *Esr1* expression and E_2_ production in an organ culture system. *Cyp19a1* and *Esr1* expression were significantly upregulated when the ovaries were cultured with activin A (Figures 9A and 9C). ELISA results showed that the E_2_ level in the culture medium was significantly higher than that in the control medium (Figure 9B), and no significant differences in *Esr2* expression were detected in the ovaries (Figure 9D). These results indicate that activin A induces CYP19A1, which increases E_2_ production, and that ESR1 is upregulated by this hormone.

## Discussion

In the present study, we investigated the molecular mechanism underlying the increased number of ovulated oocytes in OVA/hCG-injected mice compared with eCG/hCG-injected mice. Our study suggests that the induction of follicular atresia is inhibited by IAS in the ovaries, resulting in an increase in the number of surviving follicles. In addition, the study suggested that E_2_ accumulated in the follicle between 12 and 48 h after OVA injection and that the upregulation of ESR1 expression in GCs may prevent the induction of follicular atresia, resulting in an increased number of surviving follicles and ovulated oocytes.

We previously reported that the number of ovulated oocytes increased in mice administered OVA/hCG.^24^ Since the FSH level was significantly higher in the plasma of mice administered IAS^28^ and IAS neutralized inhibin activity in a rat pituitary cell culture,^18^ we hypothesized in our previous report that the high concentration of FSH in the plasma induced by the lack of negative feedback by inhibin in the pituitary promoted follicle growth and increased the ovulated oocyte number. Three-week-old mice (21- to 22-day-old) are sexually immature, and cyclic gonadotropin secretion has not yet been initiated. Thus, it is likely that FSH level in the plasma of 3-week-old mice do not increase owing to the lack of FSH secretion, even when inhibin activity is inhibited by IAS. If a high level of FSH is critical for this increase, it is likely that an increase in the number of ovulated oocytes is not observable in 3-week-old mice administered OVA/hCG. In the present study, although no significant difference in serum FSH levels was detected in mice administered eCG or OVA (Figure 1G), ovulated oocyte number increased in 3-week-old mice administered OVA/hCG (data not shown), which is not consistent with our previous hypothesis. These results led us to hypothesize that a regulatory mechanism in the ovary is involved in the increase in the number of ovulated oocytes, in addition to the possibility that high FSH levels in plasma may contribute to the increase.

**Figure 10:**
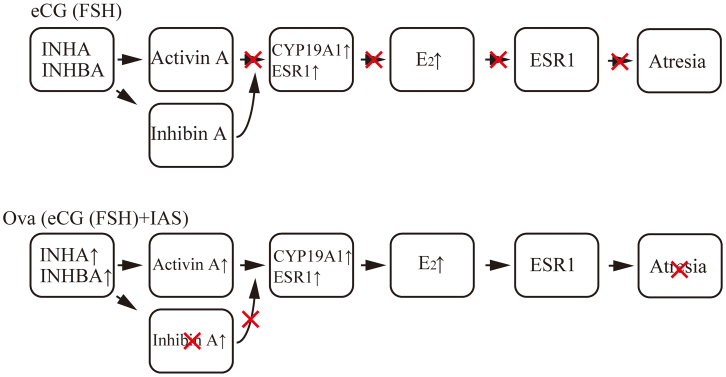
Overview of the mechanisms that induce or suppress follicular atresia in the ovaries of mice administered eCG or OVA For details, see the text.

We conclude that high E_2_ levels in the plasma and ovaries between 12 and 48 h after OVA injection and the increase in ESR1 expression in the GCs of growing follicles are critical for the suppression of follicular atresia and increased ovulated oocyte number. The rationale for this conclusion is as follows: (1) the expression of the active form of INHBA was upregulated in the ovaries of OVA-injected mice (Figure 2A), and the activin A level in the plasma of OVA-injected mice was significantly higher than that in eCG-injected mice (Figure 2C). (2) Follicles expressing INHBA were TUNEL-negative (Figure 3). (3) *Cyp19a1*/CYP19A1 and *Esr1*/ESR1 expression were significantly upregulated in the ovaries of OVA-injected mice compared with that in eCG-injected mice (Figures 4A–4C, S7A, and S7C). (4) The E_2_ levels in the plasma and ovaries of OVA-injected mice were significantly higher than those in eCG-injected mice (Figures 4D and 4E). (5) Activin A induced *Cyp19a1* expression, resulting in increased E_2_ levels in the organ culture medium (Figures 9A and 9B). (6) *Esr1* expression was upregulated in ovaries cultured with activin A (Figure 9C). (7) ESR1 expression was observed in GCs of follicles expressing INHBA (Figure 8). Based on these results and those of previous studies, a model of the mechanism of follicular atresia suppression in the ovaries of OVA-injected mice is proposed (Figure 10). INHBA is secreted by GCs and forms the homodimer, activin A. The expression level of INHBA is decreased in *Fshb* KO mice,^29^ indicating that INHBA expression is regulated by FSH. Activin A acts as a paracrine/autocrine factor in its own and neighboring follicles and induces *Cyp19a1*/CYP19A1 expression, leading to increased E_2_ production. No significant difference in serum or ovarian testosterone levels was observed compared with those of mice administered eCG (Figures 4D and 4E). Because CYP19A1 expression was upregulated in OVA-injected mouse ovaries (Figures 4A and 4C), it is suggested that the increase in the expression is crucial for the regulation of E_2_ production, rather than regulation by the balance of testosterone/E_2_ levels. Accumulated E_2_ binds to ESR1, and genes associated with follicular atresia are induced and/or suppressed. INHA is regulated by FSH and activin A, which form a heterodimer with INHBA called inhibin A.^30^ Inhibin A binds to activin A receptor type 2A and activin receptor type 2 B, which are associated with ovarian follicle development,^31^ and competitively blocks the binding of activin A to inhibit the activin signaling pathway.^32^^,^^33^^,^^34^ In this case, ESR1 expression and E_2_ production were not induced at sufficient levels to inhibit follicular atresia (Figure 10). In OVA-injected mice, the anti-INHA antibody in the OVA treatment solution binds to and neutralizes its antigen and inactivates inhibin A, which can no longer interact with its receptors and loses its inhibitory effect on the activin signaling pathway. Free activin A continues to exert its effect to produce E_2_ in GCs, resulting in the accumulation of E_2_ and increased ESR1 expression, which suppresses follicular atresia (Figure 10).

The hormonal balance between activin and inhibin is also an important regulator of follicle growth and atresia.^35^ The FSH receptor is induced by activin in follicles between the preantral and antral stages, and the FSH sensitivity in follicles is increased, resulting in the follicle growth being accelerated. This suggests that follicles in which the activin hormone level is dominant survive and enter the preovulatory stage. In OVA-injected mouse ovaries, the serum levels were increased (Figure 2C), whereas it was likely that inhibin had no or little function in the follicles because the hormone was blocked by the antibody in OVA. Therefore, the activin hormone level is likely to be dominant in many follicles between the preantral and antral stages, and many follicles may survive.

ESR1 expression was significantly increased in the ovaries 12 h after OVA injection, whereas no significant difference was detected in the ovaries 48 h after OVA injection (Figure 7). The protein was localized in theca cells at different stages of follicles in eCG-injected mice (Figure 8). A similar localization pattern was observed in OVA-injected mice. Conversely, the expression patterns detected in the GCs of antral follicles in eCG-injected mice were different from those in OVA-injected mice. Positive signals were observed in some ovarian follicles 12 h after eCG injection, whereas others were negative. The number of IHC-positive follicles tended to increase in the ovaries 12 h after OVA injection. This suggests that an increase in the number of follicles expressing ESR1 in GCs makes the difference in ERS1 expression as detected by western blotting.

The expression of *Casp 2*, *3*, and *7* was significantly decreased in the ovaries of OVA-injected mice (Figure 5). CASP2 is recognized as an initiator caspase, whereas CASP3 and 7 are effector caspases.^36^ Considering that follicles with apoptotic cells were reduced in OVA-injected mouse ovaries (Figure 1), these enzymes may play important roles in inducing the apoptosis of GCs in preantral and early antral follicles. Activin A has anti-apoptotic effects, and the expression of *Casp 3* is negatively regulated by this hormone.^37^ Activin A might be involved in the negative regulation of the *Casp* genes in OVA-injected mouse ovaries.

We observed that the number of ovulated oocytes tended to increase in mice administered E_2_ and eCG/hCG, although the difference was not significant. Conversely, mice administered DES and eCG/hCG had a significantly higher number of oocytes than the control mice (Figure 6). The terminal half-life of E_2_ in human plasma is approximately 0.5 h,^38^ whereas the biological half-life of DES in the cattle liver is 17 h.^39^ Therefore, the biological half-life of the DES in mice is likely to be much longer than that of E_2_. In addition, the binding affinity of DES to mouse estrogen receptor was approximately four times stronger than that of E_2_.^40^ The significant increase in the number of ovulated oocytes may be caused by sustained high levels of DES in the plasma and the high sensitivity of DES to the receptor. The present study showed that E_2_ levels in the plasma and ovaries 12, 24, and 48 h after OVA injection and the expression of ESR1 in the ovaries 12 h after OVA injection were significantly higher than those in mice administered eCG (Figures 4 and 7). Given that the effect of OVA on ovulation was similar to that of DES, these results support our conclusion that sustained high E_2_ levels and ESR1 expression between 12 and 48 h after OVA injection are essential for suppressing follicular atresia and increasing the number of ovulated oocytes. It may be possible that DES injection partially simulated the ovulation induction mechanism in mice administered OVA/hCG; however, the number of ovulated oocytes was much lower than that in mice administered OVA/hCG. This may be because unknown factors regulated by IAS affect ovulation induction. E_2_ is involved in the proliferation and differentiation of GCs in cooperation with insulin-like growth factor 1 (IGF-1).^41^^,^^42^ The IGF system may be required for follicle selection because IGF-1 can regulate steroidogenesis in theca cells and the proliferation and differentiation of GCs and because IGF-1 increases sensitivity to gonadotropin stimulation in early antral follicles.^43^ IGF-1 KO mice show arrested early antral follicle development, similar to the phenotype of aromatase-knockout mice, which are infertile, lack endogenous E_2_, and are arrested at the antral stage.^44^ Therefore, the number of ovulating oocytes can be increased by IGF-1 injection together with E_2_.

The results of the present study suggest that activin A, E_2_, and ESR1 are involved in the suppression of follicular atresia. In cattle and mares, models for the mechanism of follicle selection have been proposed, and studies have shown that the regulation of circulating hormone levels (e.g., FSH, gonadotropin-releasing hormone, LH, E_2_, and inhibin) in the plasma, as well as intrafollicular factors such as E_2_, IGF1, and IGFBP are essential for successful follicle selection.^10^^,^^11^ The present study suggests that mouse-dominant follicles are selected by a mechanism similar to that of mono-ovulating species. We observed high ESR1 expression in the GCs of growing INHBA-positive follicles. ESR1 expression in GCs was not observed in any of the growing follicle of mice administered eCG, whereas expression was detected in almost all follicles of mice administered OVA, suggesting that follicles with ESR1-positive GCs may survive and reach ovulation (Figure 8); however, the involvement of ESR1 in follicle selection in GCs may be mouse-specific.

Ovarian hyperstimulation syndrome (OHSS) is a serious complication associated with human-assisted reproductive technologies. High serum concentration of E_2_, increased neo-angiogenesis in ovaries, and increased vascular permeability are induced by a hyper-response to gonadotropin stimulation in patients with OHSS, resulting in an excess number of growing follicles >20.^45^ The excessively high concentration of E_2_ in the serum and growth of the excess number of growing follicles are similar in mice injected with OVA or combination of eCG and DES. Therefore, it may be possible that E_2_ is an essential hormone for follicle survival and is involved in human follicle selection.

In the present study, the comparison of the two protocols revealed part of the mechanism by which follicular atresia is inhibited by OVA. Considering that approximately 100 oocytes are ovulated in mice injected with OVA/hCG and that follicular atresia is closely related to follicle selection, the idea that follicle selection is inhibited in mice injected with OVA/hCG is likely to be reasonable. Therefore, elucidation of the overall mechanism by which the follicle selection is inhibited by OVA may provide useful information for uncovering the molecular mechanism of follicle selection. The conclusion of the present study is that follicles expressing ESR1 in GCs may be selected and reach to the preovulatory stage. Future studies are required to demonstrate that follicles are selected by the same or similar mechanisms in a natural reproductive cycle, and our findings may have a significant impact on the research of follicle selection. In addition, studies on mono-ovulating species have proposed models for the mechanism of follicle selection; however, to the best of our knowledge, only a few studies have reported follicle selection in mice. Our findings provide valuable information for investigating the molecular mechanisms underlying follicle selection in mice.

### Limitations of the study

The present study revealed part of the mechanism by which follicular atresia is inhibited by OVA and showed that follicles expressing ESR1 in GCs may be selected and reach to the preovulatory stage. Although we showed that the follicles were TUNEL-negative and that follicular atresia is likely to be suppressed in the follicles, it remains to be demonstrated whether the follicles can be selected and survive. In the present study, we investigated the mechanism using the ovaries of mice injected with OVA. Therefore, it is not clear how follicles destined to survive and those destined to induce follicular atresia are selected under natural conditions. Further studies are required to elucidate the mechanism.

## Resource availability

### Lead contact

Further information and requests for resources and reagents should be directed to and will be fulfilled by the lead contact, Katsueki Ogiwara (kogi@sci.hokudai.ac.jp).

### Material availability

Any additional information or requests to access the materials used in this study should be made to the lead contact.

### Data and code availability

• The data that support the findings of this study are available from the lead author, upon reasonable request.
• No original code was reported in this paper.
• Any additional information required to reanalyze the data reported in this paper is available from the lead contact upon request.

## Acknowledgments

This work was supported by the Grants-in-Aid for Scientific Research (18K06306 and 21K06241 to K.O.) from the Ministry of Education, Culture, Sports, Science, and Technology of Japan.

## Author contributions

E.U., M.W., Y.K., and K.O. designed and performed the experiments, prepared the figures, and analyzed the data. M.W. and K.O. drafted the manuscript. E.U. and M.W. collected the samples. N.N., T.T., and S.N. provided advice on methodology and paper preparation. All the authors have read and approved the final version of the manuscript.

## Declaration of interests

The authors declare no competing interests.

## STAR★Methods

### Key resources table

**Table:** 

REAGENT or RESOURCE	SOURCE	IDENTIFIER
**Antibodies**		

Rabbit polyclonal anti-ESR1	This paper	N/A
Goat polyclonal anti-INHA antiserum (IAS)	Takeo et al.^24^	N/A
Rabbit polyclonal anti-human INHBA	Gene Tex	Cat#GTX108405; RRID: AB_1950590
Rabbit polyclonal anti-CYP19A1	BioVision	Cat#3599-30T; RRID: AB_2088665
Mouse monoclonal anti-ACTB	Wako	Cat#010-27841; RRID: AB_2858279
Rabbit polyclonal anti-INHA	Cloud-Clone Corp.	PAA395Mu01

**Bacterial and virus strains**		

Rosetta™(DE3) Competent Cells - Novagen	Merck	Cat#70954

**Biological samples**		

Mouse ovary	This paper	N/A
Mouse serum	This paper	N/A

**Chemicals, peptides, and recombinant proteins**		

SEROTROPIN (eCG)	ASKA Animal Health	N/A
CARD HyperOva F.D.	Kyudo	N/A
Chorionic gonadotropin human	Sigma-Aldrich	Cat#CG10-1VL
Mouse recombinant ESR1	This paper	N/A
Recombinant human Activin A	Pharma Foods International Co., Ltd.	Cat#GF-001-050L
Diethylstilbestrol	Wako	Cat#559-82381
β-estradiol	Wako	Cat#052-04041

**Critical commercial assays**		

Click-iT™ Plus TUNEL Assay for *In Situ* Apoptosis Detection, Alexa Fluor™ 488 dye	Molecular Probes®	Cat#C10619
Estradiol ELISA Kit	Cyman	Cat#501890
Testosterone ELISA Kit	Cyman	Cat#582701
Mouse Activin A ELISA Kit PicoKine®	BOSTER Biological Technology	Cat#EK0302
Mouse Follicle Stimulating Hormone (FSH) ELISA Kit	MyBioSource, Inc.	Cat#MBS2700327

**Oligonucleotides**		

See Table S1		

**Recombinant DNA**		

pET30a-mESR1	This paper	N/A

**Software and algorithms**		

Image J software, version 1.53t	N/A	https://imagej.nih.gov/ij/index.html
CS Analyzer software, version 4.0	ATTO	Cat#2110030

### Experimental model and study participant details

#### Animals and housing

Jcl:ICR mice (21–22 days old) purchased from Japan SLC Inc. (Shizuoka, Japan) or bred in our laboratory were used in this study. The animals were maintained under controlled conditions of 24–25°C and a 14-h light/10-h dark cycle with free access to food and water. We checked whether ovulated oocytes were observed in the oviducts of 21-, 22-, 23-, 24-, and 25-day old mice; none of them ovulated, confirming that the mice were immature. The experimental procedures used in this study were approved by the Committee of the Center for Experimental Plants and Animals, Hokkaido University (Approval numbers: 18-0137 and 23-0079).

#### Superovulation and ovulated oocyte count

Immature female mice were administered 200 μL of PBS containing 3.75 IU of eCG alone, a mixture of eCG and 1 μL of EtOH, or a mixture of eCG and 1 μL of either E_2_ solution (2 ng) (Wako Pure Chemicals, Osaka, Japan) or DES solution (2 ng) (Wako). At 12 h after the injection, 200 μL of PBS containing 1 μL of either E_2_ solution (2 ng) or DES solution (2 ng) was injected into the mice. At 36 h after the last injection, 5 IU of hCG (Sigma-Aldrich) was injected into the mice. Sixteen hours after the hCG injection, the mice were sacrificed by cervical dislocation and their oviducts were collected. The ampulla of the oviduct was severed with a dissecting needle and cumulus-oocyte complexes (COCs) were harvested. The COCs were incubated with hyaluronidase (Wako) at 4°C for 16 h, and then the number of ovulated oocytes was counted.

#### Organ culture

Whole ovaries isolated from immature female mice were cultured in Dulbecco’s modified Eagle medium (Wako) supplemented with 1× penicillin-streptomycin-amphotericin B suspension (Wako) and 1× L-glutamine solution (Wako), with or without 50 ng/mL recombinant human Activin A (rActivin A) (Pharma Foods International Co., Ltd., Kyoto, Japan). After 24 h of culture, ovaries and culture media were collected and used for qRT-PCR and E_2_ ELISA, respectively.

### Method details

#### Ovaries and sera sampling

Mice were administered 3.75 IU of eCG (ASKA Animal Health, Tokyo, Japan) or 200 μL of CARD HyperOva (OVA; Kyudo, Saga, Japan), which is composed of eCG and IAS. At 12, 24, and 48 h after injection, the ovaries were isolated and sera were collected and stored at -80°C, and -20°C, respectively, until further use.

#### rESR1 and its antibody preparation

Mouse recombinant ESR1 (rESR1) was produced using an *Escherichia coli* expression system as previously described.^46^ Briefly, the coding region of ESR1 was amplified by RT-PCR with cDNA from ovaries isolated from mice 48 h after eCG injection using KOD-Plus-Neo DNA polymerase (Toyobo, Osaka, Japan). Primer pairs used in this study are listed in Table S1. The PCR product was phosphorylated, gel-purified, and ligated into a pET30a vector that had been digested with *Eco*RV. The resulting vector was confirmed by sequencing. The vector was transformed into *E. coli* Rosetta strain and the expression of rESR1 was induced. After purification, antibodies were generated in rabbits using the purified protein by a custom antibody production service (Eurofins Genomics K. K., Tokyo, Japan). The specific antibodies were purified as previously described.^46^

#### TUNEL assay and immunohistochemistry

Paraffin sections (5-μm thick) were prepared as previously described.^47^ Briefly, the ovaries were fixed in Bouin's solution (Wako) for 17 h and embedded in paraffin. The sections were dewaxed, hydrated using a graded series of ethanol solutions, rinsed in pure water for 5 min, and used for TUNEL assay and immunohistochemistry. The TUNEL assay was performed using a Click-iT™ Plus TUNEL Assay for *In Situ* Apoptosis Detection, Alexa Fluor™ 488 dye (Molecular Probes®, Thermo Fisher Scientific, Waltham, MA) following the manufacturer’s instructions. The area of the positive cells and the whole area of the follicle were measured using Image J software, version 1.53t (https://imagej.nih.gov/ij/index.html).

Immunohistochemistry was performed following previous method.^46^ Briefly, sections were boiled in 10 mM sodium citrate (pH 6.0) for 45 min, washed with phosphate-buffered saline (PBS), and incubated in PBS containing 3% H_2_O_2_ for 5 min. The samples were then blocked and incubated with an anti-human INHBA antibody (Genetex, CA, USA), anti-INHA antibody,^24^ or a purified anti-mouse ESR1 antibody. After washing with PBS, the sections were incubated with Dako EnVision+System-HRP labeled polymer anti-rabbit (for INHBA and ESR1) (Agilent Technologies, Inc. Santa Clara, CA, USA), or rabbit anti-goat IgG antibody, HRP conjugate (for INHA) (Merck & Co. Inc., Raymond, NJ, USA) diluted in PBS containing 0.3% Tween 20 before the signals were detected. Hematoxylin and eosin staining was performed to observe the histological morphology of the ovaries of mice injected with eCG or OVA (Figure S2).

#### Western blotting

The ovaries were sonicated on ice in 50 mM Tris-HCl (pH 8.0) containing a 1 × protease inhibitor mixture (Wako). After centrifugation at 13,000 × g for 10 min at 4°C, the supernatant was collected and used for western blotting for INHA, INHBA, and CYP19A1, and E_2_ purification. The precipitated fraction was boiled in 50 mM Tris-HCl (pH 8.0) containing 1% SDS for 20 min and centrifuged at 13,000 × g for 10 min. The resulting supernatant was collected and used for western blot analysis of ESR1. The protein concentration was determined using a BCA Kit (Thermo Fisher Scientific). Western blotting was performed as previously described^46^ except that anti-CYP19A1 (Bio-vision, Doha, Qatar), anti-INHA, anti-human INHBA, purified anti-mouse ESR1, and anti-ACTB antibodies (Wako) were used for detection. To prepare an absorbed antibody for ESR1, the antibody was pre-incubated with its antigen (10 μg) for 1 h at room temperature and used for the reaction. The optical band density was measured using CS Analyzer software (version 4.0; ATTO, Tokyo, Japan). As a negative control, western blotting was performed using normal rabbit, goat, or mouse IgG as the primary antibody.

#### E_2_ and testosterone purification

E_2_ and testosterone were purified from serum and ovarian extracts using hydrophobic column chromatography (Agilent Technologies). The column was then rinsed with 5.0 mL of dichloromethane and 2.5 mL of methanol. After washing with 2.5 mL of Milli-Q water, 1 mL of sample solution was added to the column. The column was then washed with 2.5 mL of Milli-Q water and rinsed with 10.0 mL of 50% methanol. After drying the column for 10 min, the bound materials were eluted with 5.0 mL of dichloromethane. The dichloromethane in the eluted solution was evaporated by centrifugation at 2000 × g at room temperature. The dried sample was dissolved in 5 μL of methanol and then 40 μL of Milli-Q water and 5 μL of 10× ELISA buffer provided in the competitive Estradiol ELISA Kit (Cayman Chemical, MI) or Testosterone ELISA Kit (Cayman). The samples were stored at -20°C until use.

#### ELISA

For Inhibin A and/or Inhibin B ELISA, a rabbit anti-inhibin α subunit antibody (Cloud-Clone, TX, USA) was diluted 1:100 in 100 μL of PBS, and it was incubated in an anti-rabbit IgG-coated plate (Thermo Fisher Scientific) at room temperature for 1 h. The wells were washed three times with PBS and incubated with PBS containing 1% bovine serum albumin at room temperature for 1 h. The plate was washed with PBS three times and 100 μL of the serum samples were added to the wells. After incubation at room temperature for 1 h, the wells were washed with PBS three times and then incubated with a goat anti-inhibin α subunit antibody diluted 1:200 in 100 μL of PBS at room temperature for 1 h. The wells were washed with PBS three times and then with a horseradish peroxidase-conjugated anti-goat antibody (GE Healthcare) diluted 1:5000 in 100 μL of PBS at 4°C for 16 h. The plate was then washed with PBS three times, 100 μL substrate solution (37 μM o-phenylenediamine, 3% v/v H2O2 in phosphate/citric acid buffer; 0.1 M citric acid, and 0.2 M Na2HPO4, pH 5.0) was added to the wells, and that plate was incubated at room temperature for 10 min in the dark. The absorbance of each solution was measured at 492 nm using a SmartSpec 3000 spectrophotometer. Each sample was assayed in duplicates.

Activin A, FSH, E_2_, and testosterone levels were measured using a Mouse Activin A ELISA Kit PicoKine® (BOSTER Biological Technology, Pleasanton, CA), a Mouse FSH ELISA Kit (MyBioSource, Inc., San Diego, CA), an Estradiol ELISA Kit, and a Testosterone ELISA Kit, respectively, according to the manufacturer’s instructions. Absorbance was measured at 414 nm (for E_2_ and testosterone) or 450 nm (for FSH) using a spectrophotometer (SmartSpec 3000). The detection limit of Activin A, FSH, E_2_, and testosterone was 10.0 pg/mL, 0.96 ng/mL, 6.0 pg/mL, and 3.0 pg/mL, respectively. Each sample was assayed in duplicates.

#### Quantitative PCR (qPCR)

Total RNA was isolated from the ovaries using Isogen I (Nippon Gene, Tokyo, Japan), following the manufacturer’s instructions. Reverse transcription was conducted using a ReverTra Ace® qPCR RT Master Mix with gDNA Remover (Toyobo) according to the manufacturer’s instructions. The qPCR was performed as previously described.^48^ KOD SYBR qPCR Mix (Toyobo) or KAPA Fast qPCR Kit (Nihon Genetics Co., Ltd., Tokyo, Japan) was used for PCR analysis. Gene expression levels in the ovaries were normalized to those of cytoplasmic actin (Actb). The primers used for qPCR are listed in Table S1.

### Quantification and statistical analysis

Experiments were repeated 4-20 times, and the data are presented as mean ± SEM. Statistical evaluation was performed with Excel software using Student’s t-test or one-way analysis of variance (ANOVA) followed by the Tukey–Kramer test. Statistical significance was set at *p* < 0.05. Representative results of at least three independent experiments are shown for the western blotting, immunohistochemistry, and TUNEL assays. Relative quantification was analyzed using Image J software, version 1.53t (https://imagej.nih.gov/ij/index.html) or CS Analyzer software (version 4.0; ATTO). The statistical details of experiments are described in the legends.
