# Supplementary material for: 17β-estradiol and estrogen receptor alpha protect mouse ovarian follicle development by repressing atresia
Source: iScience. 2025 Jan 20;28(2):111846. doi: 10.1016/j.isci.2025.111846 (PMC11841210; doi:10.1016/j.isci.2025.111846)
Supplement: Document S1. Figures S1 and S2 and Table S1 [file mmc1.pdf]

iScience, Volume *28*

Supplemental information

17β-estradiol and estrogen receptor alpha protect mouse ovarian follicle development by repressing atresia

Eri Ueno, Mitsuya Watanabe, Yoshiko Kondo, Naomi Nakagata, Toru Takeo, Satohiro Nakao, and Katsueki Ogiwara

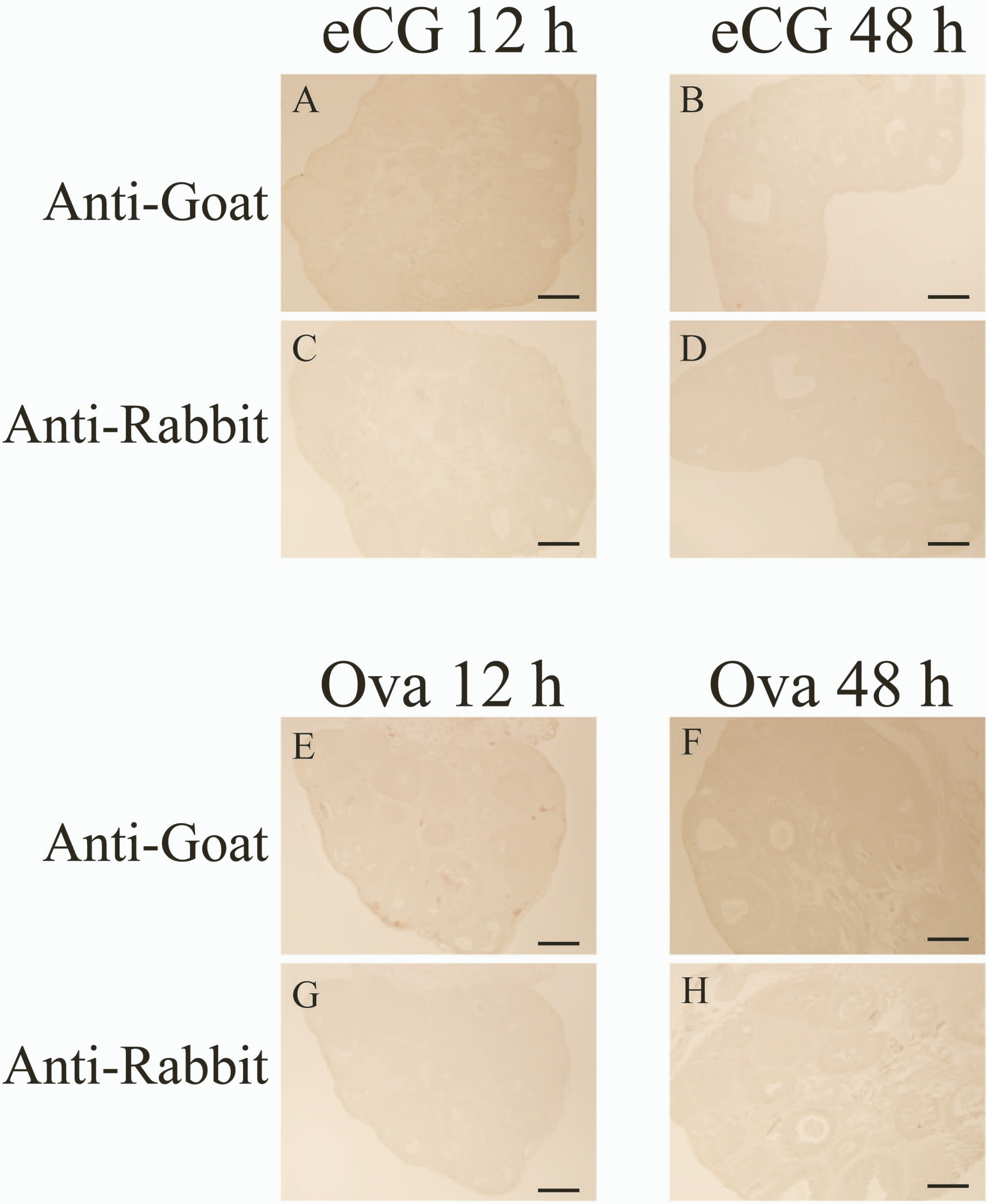

Figure S1. Negative control for immunohistochemistry.
Immunohistochemistry was performed with anti-goat, or anti-rabbit normal IgG as the primary antibody using sections prepared from ovaries harvested 12 and 48 h after the mice were administered eCG (left panels) or OVA (right panel). Bars = 100 μm.

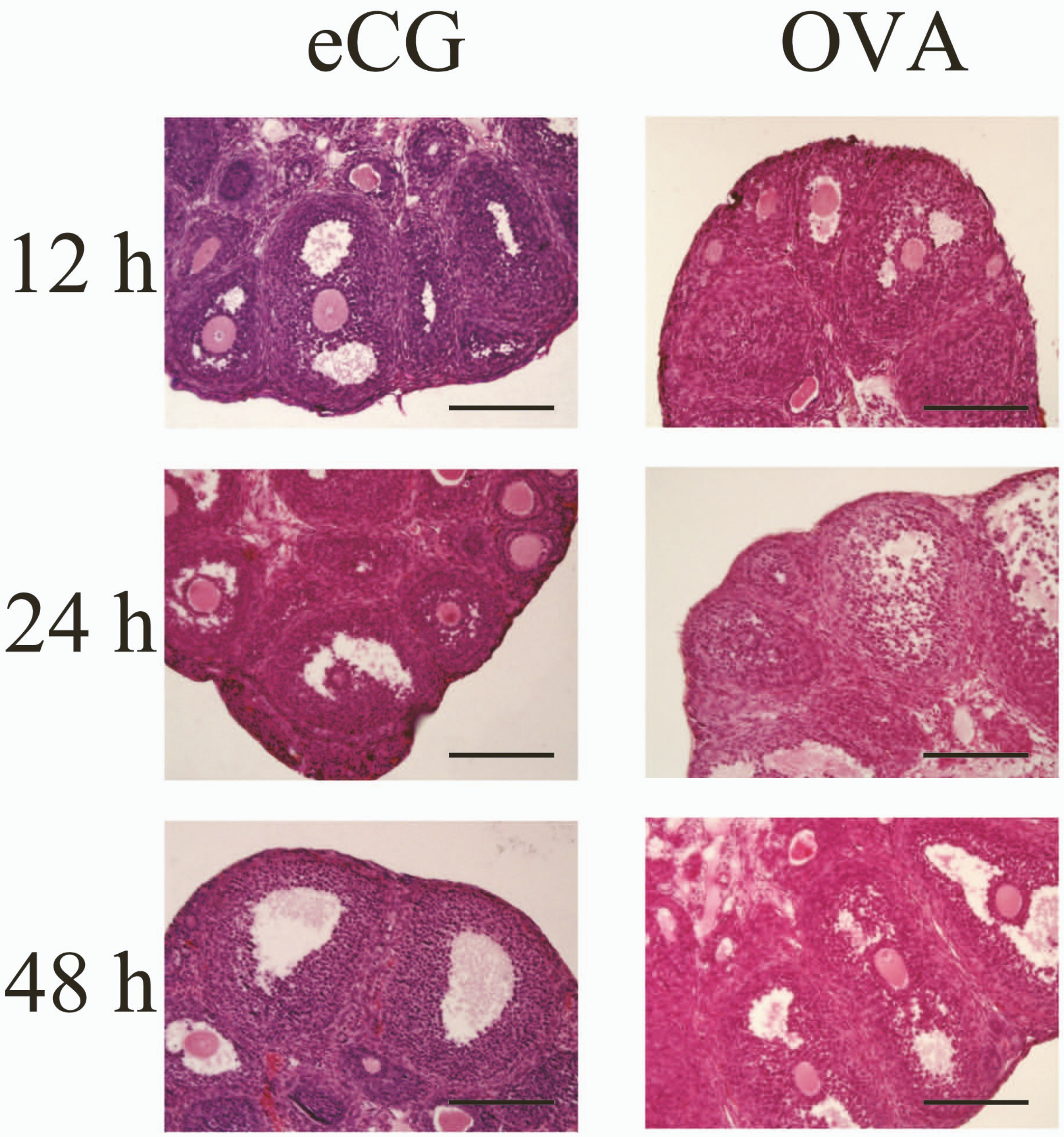

Figure S2. Images of ovarian sections of mice administered eCG or OVA. Sections prepared from ovaries harvested 12, 24 and 48 h after the mice were administered eCG (left panels) or OVA (right panels) were stained with hematoxylin and eosin. Bars = 100 μm.

Table S1. Primers used in this study.

Primer name	gene	Sequence	Accession No.
Real-time PCR			
Esr1 ss	*Esr1*	5′-AATGGCCTTGCTGCACCAGA-3′	AB560752
Esr1 as	*Esr1*	5′-TGCTGTTGTCCACGTATACC-3′	AB560752
Esr2 ss	*Esr2*	5′-TAGGCATTCCACCTGCCCGT-3′	BC145329
Esr2 as	*Esr2*	5′-GGAAGTAAGGCTTGATGGTG-3′	BC145329
Star ss	*Star*	5′-GCATACTCAACAACCAGGAAGG -3′	L36062
Star as	*Star*	5′-AGAGTCTGTCCATGGGCTGGTC-3′	L36062
Cyp17a1 ss	*Cyp17a1*	5′-CCCTGTTCAGGGATGACCAGAA-3′	BC064793
Cyp17a1 as	*Cyp17a1*	5′-TGAAGATGAGCGTAGACAGATC-3′	BC064793
Hsd17b1 ss	*Hsd17b1*	5′-TTTGTGCGAGAGTCTGGCGATCC-3′	BC125659
Hsd17b1 as	*Hsd17b1*	5′-CTGGGCATCTGCTCGTTCCAGA-3′	BC125659
Cyp11a1 ss	*Cyp11a1*	5′-GTGGCCTATCACCAGTATTATC-3′	BC068264
Cyp11a1 as	*Cyp11a1*	5′-ACCTCTTGGTTTAGGACGATTC-3′	BC068264
Hsd3b1 ss	*Hsd3b1*	5′-TTCCCTAAGCCCCTGCTCAGAG-3′	BC052659
Hsd3b1 as	*Hsd3b1*	5′-GGATTTTACCAAAGGCAAGA-3′	BC052659
Cyp19a1 ss	*Cyp19a1*	5′-TATAATGTCACCATCATGGTCC-3′	BC103670
Cyp19a1 as	*Cyp19a1*	5′-GCCAGGACCTGGTATTGAAGA-3′	BC103670
Casp1 SS	*Casp1*	5′-TCAGAAAGGTTCGATTTTCA-3′	NM_009807
Casp1 AS	*Casp1*	5′-AGAATGAACTGGATTCTTCG-3′	NM_009807
Casp2 SS	*Casp2*	5′-CTGTAGTACTCTGTGCCAGC-3′	D28492
Casp2 AS	*Casp2*	5′-AAGAGAAGTCTCCATTGTGC-3′	D28492
Casp3 SS	*Casp3*	5′-TGTTATGCCAAATGAGAAAG-3′	Y13086
Casp3 AS	*Casp3*	5′-CAACTACCTGATGTCGAAGT-3′	Y13086
Casp6 SS	*Casp6*	5′-AAGCTGCATTTCTGTCCCAA-3′	NM_009811
Casp6 AS	*Casp6*	5′-TGAAGCAGTTTACATTGATG-3′	NM_009811
Casp7 SS	*Casp7*	5′-TTCATCTGATGACTTCATGC-3′	Y13088
Casp7 AS	*Casp7*	5′-AGGTCATGCTCACAGCATGC-3′	Y13088
Casp8 SS	*Casp8*	5′-TGGCGTGAACTATGACGTGA-3′	AF067834
Casp8 AS	*Casp8*	5′-GTGAACTGTGGAGAGCACAC-3′	AF067834
Casp9 SS	*Casp9*	5′-ATGCTGTGTCAAGTTTGCCT-3′	AB019600
Casp9 AS	*Casp9*	5′-CCATCCAAGGTCTCGATGTA-3′	AB019600
Casp11 SS	*Casp11*	5′-ACCTTGACGAGATATTTCTA-3′	Y13089
Casp11 AS	*Casp11*	5′-CAGGAATAGAATGTGATCCT-3′	Y13089

Table S1. (continue) Primers used in this study.

Primer name	gene	Sequence	Accession No.
Real-time PCR			
Casp12 SS	*Casp12*	5′-TTTCCAAACTCATTGACTGC-3′	AY675224
Casp12 AS	*Casp12*	5′-GCGTGTCATGGATACTCTCT-3′	AY675224
Recombinant protein			
ESR1 pET SS	*Esr1*	5′-ATGACCATGACCCTTCACAC-3’	AB560752
ESR1 pET AS	*Esr1*	5′-TCAGATCGTGTTGGGGAAGC-3’	AB560752
